# *Vitis vinifera* Extract Ameliorate Hepatic and Renal Dysfunction Induced by Dexamethasone in Albino Rats

**DOI:** 10.3390/toxics5020011

**Published:** 2017-04-11

**Authors:** Nabil A. Hasona, Ahmed A. Alrashidi, Thamer Z. Aldugieman, Ali M. Alshdokhi, Mohammed Q. Ahmed

**Affiliations:** 1 Biochemistry Department, College of Medicine, University of Hail, Hail 2440, Saudi Arabia; 2 Chemistry Department, Biochemistry Division, Faculty of Science, Beni-Suef University, Beni-Suef 62511, Egypt; 3 College of Medicine, University of Hail, Hail 2440, Saudi Arabia; celesa7med@gmail.com (A.A.A.); thamer265@hotmail.com (T.Z.A.); aboshdkh@gmail.com (A.M.A.); 4 Pharmacology Department, College of Medicine, University of Hail, Hail 2440, Saudi Arabia; clinical.cology@gmail.com

**Keywords:** alteration, hepatic, dexamethasone, grape seed, catalase, glucose-6-phosphate dehydrogenase, GSH

## Abstract

This study was conducted to evaluate the biochemical effects of grape seed extract against dexamethasone-induced hepatic and renal dysfunction in a female albino rat. Twenty-eight adult female rats were divided randomly into four equal groups: Group 1: animals were injected subcutaneously with saline and consider as normal control one. Group 2: animals were injected subcutaneously with dexamethasone in a dose of 0.1 mg/kg body weight. Group 3: animals were injected subcutaneously with 0.1 mg/kg body weight of dexamethasone, and then treated with a grape seed extract in a dose of 200 mg/kg body weight by oral gavage. Group 4: animals were injected subcutaneously with 0.1 mg/kg body weight of dexamethasone, and then treated with a grape seed extract in a dose of 400 mg/kg body weight by oral gavage. After 4 weeks, serum alanine aminotransferase (ALT), aspartate aminotransferase (AST) activities, albumin, uric acid, creatinine, and glucose levels were assayed. Hepatic reduced glutathione (GSH), total protein content, and catalase and glucose-6-phosphate dehydrogenase activities were also assayed. Dexamethasone administration caused elevation of serum levels of glucose, uric acid, creatinine, ALT, AST activities, and a decrease in other parameters such as hepatic glutathione, total protein levels, and catalase enzyme activity. Treatment with *Vitis vinifera* L. seed extract showed a significant increase in the body weight of rats in the group treated with *Vitis vinifera* L. seed extract orally compared with the dexamethasone control group. An increase in GSH and catalase activity in response to oral treatment with *Vitis vinifera* L. seed extract was observed after treatment. Grape seed extract positively affects glucocorticoid-induced hepatic and renal alteration in albino rats.

## 1. Introduction

Recently, the trends in traditional and complementary medicines for patients are becoming more interested in medicinal plants [1]. Some medicinal plants may exert promising pharmacological properties and improve the effectiveness of conventional medications as complementary agents [2].

*Vitis vinifera* (Grape) is one of the most consumed fruits globally. *V. vinifera* possesses a wide range of pharmacological activities due to its rich polyphenol ingredients, most of which are contained in its seeds [3]. Grape seed extract comprises flavonoids such as proanthocyanidins, which are potent antioxidants and stimulate many health-promoting effects [3]. Grape seed extract has a wide range of pharmacological and remedial impacts such as antioxidative, anti-inflammatory, and additionally having cardioprotective, hepatoprotective, and neuroprotective effects [4].

Previous studies have also shown that grape seed extracts repress enzymes responsible for free radical formation and also have antimutagenic and anticarcinogenic properties [5,6,7].

Glucocorticoids are among the most utilized medications around the world, and they are effective medications for a multitude of inflammatory and immunological disorders. Despite its therapeutic activity, high doses and long-term utilization of these medications are concomitant with serious side effects, such as diabetes mellitus, liver disorder, cardiovascular events, hypertension, dyslipidemia, and osteoarthritis [8,9].

Dexamethasone is a potent synthetic glucocorticoid medication for multitudinous inflammatory and immunological disorders [9]. Similar to other glucocorticoids, high doses and long-term utilization of dexamethasone are associated with negative side effects [10].

In the current study, we hypothesized that *V. vinifera* seed extract was capable of preserving activity levels of metabolizing enzymes and preventing glucocorticoid side effects. We further hypothesized that the seed extract was able to reduce free radicals and enhance antioxidant activity.

## 2. Materials and Methods

### 2.1. Grape Seed Extract Preparation

Grape seeds were removed from fresh fruits purchased from a local market in Hail City, KSA and thoroughly washed, and were then dried. The dried seed were ground to powder form. The powder was soaked for 24 h in an appropriate volume of Millipore distilled water to obtain 200 mg/mL solution. The solution was filtered by using 0.22 µm sterile filters with the help of a vacuum pump, then stored in a brown bottle at 4 °C until use. 

### 2.2. Drugs and Chemicals

Dexamethasone (4 mg/mL, EIPICO, 10^th^ of Ramadan City, Egypt) was used in the study. Before administration, dexamethasone was diluted with normal saline. All chemicals used in our study were of analytical or reagent grade. 

### 2.3. Animals

The twenty-eight female albino rats of the Wistar Strain weighing 175–205 g used for this study were obtained from the animal house of the college of pharmacy, King Saud University, Riyadh, KSA. They were housed at 26 ± 2 °C, 12 h:12 h light:dark cycle. Animals were provided with a standard diet and water ad libitum. Rats were allowed to acclimate to laboratory conditions for 7 days prior to dosing. The experimental work on female rats was carried out in accordance with the Institutional Scientific and Research Ethics Committee, College of medicine, Hail University, KSA (EC Ref No.: EC-014, 15 December 2016).

### 2.4. Animal Groups

After the acclimation period, 28 female albino rats were randomly divided into four groups of seven each, as follows: Rats of group I were injected subcutaneously with normal saline only and considered intact control. Animals of group II served as the dexamethasone-control and were injected subcutaneously with dexamethasone (0.1 mg/kg/day) [11]. Rats of group III were injected with dexamethasone and treated with grape seed extract (200 mg/kg BW) by oral gavage [12]. Animals of group IV were injected with dexamethasone and treated with grape seed extract (400 mg/kg BW) by oral gavage [12]. 

All experimental animals were treated three times per week for four consecutive weeks, and the administration with dexamethasone and grape seed extract were done between 7.30 a.m. and 9.00 a.m.

After the end of experimental duration (30 days), rats were fasted for 12 h and sacrificed under light diethyl ether anaesthesia. Blood samples were collected from each rat; serum was drawn after centrifugation at 3500 rpm for 10 min at 4 °C. The sera were kept in a deep freezer (−20 °C) for further biochemical assays.

During the experimental period, body weight changes of female rats were recorded weekly. Liver and kidney of rats from each group were quickly removed, cleaned, weighed, and used for biochemical studies. Then, relative weight was calculated.

### 2.5. Biochemical Examination

The glucose level in serum was assayed using the glucose oxidase method [13]. Serum activities of aspartate aminotransferase (AST), and alanine aminotransferase (ALT) were assayed according to Reitman and Frankel [14] using reagent kits purchased from Kashef diagnostic company (Jeddah, Saudi Arabia). Serum albumin level was assayed by Bromo cresol green (Kashef diagnostic company, Jeddah, Saudi Arabia). Serum levels of creatinine and uric acid were assayed according to the methods of [15,16], respectively.

Hepatic glucose-6-phosphate dehydrogenase activity (G6PDH) was assayed according to [17], based on the conversion of NADP^+^ to NADPH and followed by monitoring the change in absorbance at 340 nm.

The hepatic catalase activity was assayed according to Cohen et al. [18], based on the ability of catalase to induce the H_2_O_2_ decomposition.

The hepatic content of reduced glutathione (GSH) was assayed by the spectrophotometric technique according to Sedlack and Lindsay [19].

### 2.6. Statistical Analysis

The data analysis was carried out using statistical software SPSS 18.0 (SPSS Inc., Chicago, IL, USA). Data are presented as a mean ± standard deviation. Statistical analyses were performed using one-way ANOVA, and means were compared using Duncan’s multiple range test as a post hoc test at the 5% probability level. A *p*-value <0.05 was considered statistically significant. 

## 3. Results

Table 1 shows the effect of dexamethasone administration on the body weight gain of female albino rats and relative liver weight. One way ANOVA test showed a highly significant effect of treatment on the body weight gain in the different animal groups (F = 61.88, *p* < 0.001). The control group showed a mean weight gain of 32.64 ± 10.21 g. Dexamethasone administrations decreased this value significantly to −60.87 ± 17.40 g. Treatment with 200 mg/kg BW grape seed extract after dexamethasone administration significantly decreased the weight gain to −9.86 ± 16.01 g. Treatment with 400 mg/kg BW GSE after dexamethasone administration increased the weight gain to 7.30 ± 6.57 g. Additionally, the relative liver weight of animals in the four groups was significantly different, as shown in Table 1. 

Table 2 shows the effect of the administration of dexamethasone on the liver function tests. For ALT, one-way ANOVA test showed a highly significant effect of treatment in the different animal groups (F = 102.94, *p* < 0.001). The control group showed a mean ALT enzyme activity of 55.57 ± 8.92 U/L. Administration of dexamethasone elevated this value significantly to 124.29 ± 6.52 U/L. Treatment with 200 mg/kg BW GSE significantly decreased the enzyme activity to 79.43 ± 4.50 U/L, whereas with 400 mg/kg BW GSE, ALT activity reduced to 69.71 ± 9.86 U/L. For AST, a very similar effect was shown (F = 167.17, *p* < 0.001). For albumin, one-way ANOVA test showed a highly significant effect of administration in the different animal groups (F = 55.87, *p* < 0.001). The control group showed a mean albumin level of 28.29 ± 2.98 g/L. Administration of dexamethasone decreased this value significantly to 11.34 ± 1.76 g/L. Treatment with 200 mg/kg BW GSE significantly elevated the albumin level to 18.07 ± 1.88 g/L, whereas with 400 mg/kg BW GSE, albumin level increased to 24.17 ± 3.44 g/L, as shown in Table 2.

Table 3 shows the effect of administration of dexamethasone on the fasting glucose, uric acid, and creatinine levels. For glucose, one-way ANOVA test showed a highly significant effect of treatment in the different animal groups (F = 72.01, *p* < 0.001). The control group showed a mean glucose level of 4.79 ± 0.53 mmol/L. Administration of dexamethasone elevated this value significantly to 9.37 ± 0.98 mmol/L. Treatment with 200 mg/kg BW GSE significantly decreased the glucose level to 6.64 ± 0.25 mmol/L, whereas with 400 mg/kg BW GSE, glucose level reduced to 5.71 ± 0.47 mmol/L. For uric acid and creatinine level, a very similar effect was shown (Table 3). 

**Figure 1:**
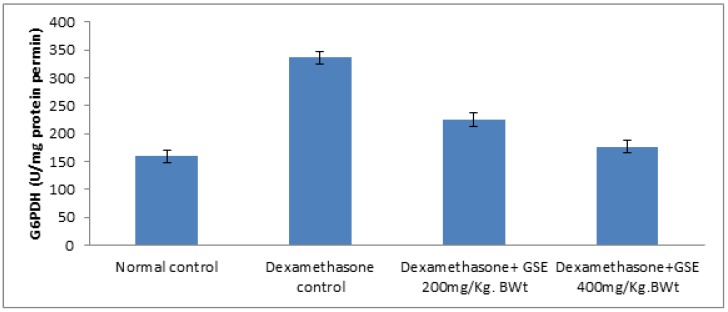
Effect of grape seed extract (GSE) on hepatic activity of glucose-6-phosphate dehydrogenase (G6PDH) in albino rats injected with dexamethasone (*n* = 7).

Regarding hepatic activity of glucose-6-phosphate dehydrogenase, our findings revealed that the grape seed extract administration to female albino rats produced profound and significant reduction in the hepatic glucose-6-phosphate dehydrogenase, as shown in Figure 1.

With respect to the oxidative stress markers, hepatic catalase enzyme, GSH, and total protein content were studied. For hepatic catalase enzyme activity, one-way ANOVA test showed a highly significant difference between the different animal groups (F = 111.01, *p* < 0.001). The control group showed enzyme activity of 0.88 ± 0.05 UI/mg protein; enzyme activity reduced to 0.42 ± 0.04 UI/mg proteins after dexamethasone administration. Treating the animals with 200 mg/kg GSE increased the catalase enzyme activity to 0.54 ± 0.03 UI/mg protein, whereas treatment with 400 mg/kg GSE increased the enzyme activity to 0.70 ± 0.07 UI/mg protein. Hepatic GSH and total protein content showed the same trend as catalase enzyme activity (Table 4).

## 4. Discussion

Dexamethasone is a synthetic glucocorticoid indicated for treating inflammatory and autoimmune syndromes. However, dexamethasone therapy is associated with a variety of side effects, including liver and kidney dysfunction. The current study reveals the protection and the dose–response effect conferred by grape seed extract (*Vitis vinifera*) against dexamethasone-induced oxidative stress in experimental female albino rats. 

The administration of dexamethasone significantly decreased the body weight gain and relative liver and kidney weights at the end of the experimental period. These results were in accordance with the findings of [20,21]. The adverse effects of glucocorticoid treatment are well-known, including the induction of insulin resistance and some metabolic disorders like hyperleptinemia, loss of appetite, and weight loss with a higher level of blood glucose and triglycerides [22,23].

Grape seed extract treatment repressed dexamethasone-induced weight loss and showed a conversely marginal increase in body weight. The improvement of body weight gain after grape seed extract may be due to the increase in the sensitivity to insulin and the resultant increase in the glucose uptake [24].

The liver is structurally heterogeneous, and therefore executes various important functions, such as detoxification. Liver enzymes such as AST and ALT are among the marker enzymes for liver function and integrity [25,26] which are usually elevated in the manifestation of acute hepatotoxicity or mild hepatocellular injury. In our study, administration of dexamethasone led to a significant elevation in AST and ALT activities, and conversely to a decrease in albumin level. These results are in accordance with [27,28].

Elevation of AST and ALT enzyme is attributed to the damaged structural integrity of the liver [25]. Additionally, the decreased level of albumin after dexamethasone administration may be due to its damaging effect on DNA and RNA. Treatment with grape extract to dexamethasone-exposed rats produced significant improvement in liver functions, indicating the beneficial role of grapes to counteract the dexamethasone-induced liver dysfunctions. These results are in accordance with [29,30].

In the current study, the dexamethasone-administered rats showed a significant elevation in blood glucose level, which agrees with previous studies [21]. A dose-dependent decrease in glucose levels was observed in grape seed extract-treated rats compared to the dexamethasone control. 

Many factors clarify hyperglycemia after dexamethasone administration, such as decreased insulin sensitivity, attenuated pancreatic α- and β-cell functions, and augmented hepatic gluconeogenesis [31].

It was also observed that rats treated with dexamethasone showed a significant elevation in serum uric acid and creatinine levels. Creatinine is thought to be a dependable indicator of how well the kidneys are filtering out toxins. Treatment of rats with grape seed extract resulted in a significant improvement in uric acid and creatinine levels. These findings agree with [32], who showed the ameliorative effects of grape seed on serum kidney functions of paracetamol-induced hepatotoxicity in rats.

In the current study, dexamethasone induced increased oxidative stress, which is presented by a significantly decreased hepatic GSH content and decreased catalase activity. Oxidative stress has been stated as a major cause of dexamethasone-induced liver injury and extreme production of free radicals. These findings agree with results reported by [21,33].

The protection of grape seed extract against oxidative stress was measured by detecting glutathione and catalase (in liver tissues). Moreover, GSE (200 and 400 mg/kg) significantly increased hepatic GSH content and catalase activity in a dose-dependent manner to near the normal levels. Accordingly, this is because grape seeds have antioxidant and free radical scavenging activities, therefore protecting against oxidative stress and replenishing the reduced glutathione content. These findings agree with results reported by [34,35], who reported that oral administration of grape seed extract ameliorated and enhanced the antioxidant defense against Ehrlich solid tumor-induced oxidative stress in mice.

The potential beneficial role of grape seed extract is in preventing oxidative stress-mediated damage and strengthening antioxidant defense mechanisms, with an increase in the antioxidant status of animals. These actions are due to the content of phytochemicals like polyphenolic compounds such as procyanidins and proanthocyanidins, which have a powerful free radical scavenging effect by inhibiting advanced glycation end product (AGE) formation, which helps reverse the effects on lipid peroxidation level and the activities of antioxidant enzymes.

Glucose-6-phosphate dehydrogenase is a crucial enzyme of the pentose phosphate pathway that catalyses key steps in the regulation of redox balance and in anabolic processes [36]. Our results demonstrate that the hepatic activity of glucose-6-phosphate dehydrogenase was significantly elevated after administration with dexamethasone in rats. Our findings are consistent with other authors [37]. Overexpression and up-regulation of glucose-6-phosphate dehydrogenase in liver tissue may promote oxidative stress, which is a major cause of dexamethasone-induced liver injury. Similarly, authors in [38,39] reported that up-regulation of glucose-6-phosphate dehydrogenase activity increases oxidative stress, which leads to functional defects in the tissues. The ameliorative effects of grape seed extract significantly decrease hepatic glucose-6-phosphate dehydrogenase activity in a dose-dependent manner to near-normal levels. This is because grape seeds have antioxidant and free radical scavenging activities.

## 5. Conclusions

Our findings showed that dexamethasone is capable of triggering marked oxidative stress. Supplementation with grape seed exerted undeniable ameliorative and therapeutic action against dexamethasone-induced oxidative stress.

## Figures and Tables

**Table 1:** Effect of grape seed extract on body weight (BW) gain and relative liver and kidney weight in albino rats injected with dexamethasone (*n* = 7).

Groups	Body Weight Gain (g)	Relative Liver Weight (g/100 BW)	Relative Kidney Weight (g/100 BW)
Group (1)	32.64 ^d^ ± 10.21	3.84 ^b^ ± 0.27	0.68 ± 0.05
Group (2)	−60.87 ^a^ ± 17.40	4.68 ^c^ ± 1.23	1.09 ± 0.19
Group (3)	−9.86 ^b^ ± 16.01	2.99 ^a^ ± 0.41	0.94 ± 0.15
Group (4)	7.30 ^c^ ± 6.57	3.36 ^a,b^ ± 0.36	0.81 ± 0.08
F-ratio	61.88	7.88	12.34
*p*-value	0.00	0.00	0.00

The different letters indicate statistically different means according to Duncan multiple range test.

**Table 2:** Effect of grape seed extract on liver function tests in albino rats injected with dexamethasone (*n* = 7).

Groups	ALT (U/L)	AST (U/L)	Albumin (g/L)
Group (1)	55.57 ^a^ ± 8.92	80.14 ^a^ ± 4.85	28.29 ^d^ ± 2.98
Group (2)	124.29 ^d^ ± 6.52	175.29 ^c^ ± 12.26	11.34 ^a^ ±1.76
Group (3)	79.43 ^c^ ± 4.50	123 ^b^ ± 6.68	18.07 ^b^ ± 1.88
Group (4)	69.71 ^b^ ± 9.86	114.86 ^b^ ± 6.36	24.14 ^c^ ± 3.44
F-ratio	102.94	167.17	55.87
*p*-value	0.00	0.00	0.00

The different letters indicate statistically different means according to Duncan multiple range test. ALT is alanine aminotransferase; AST is aspartate aminotransferase.

**Table 3:** Effect of grape seed extract on fasting blood glucose, uric acid, and creatinine levels in albino rats injected with dexamethasone (*n* = 7).

Groups	Fasting Blood Glucose (mmol/L)	Uric Acid (µmol/L)	Creatinine (µmol/L)
Group (1)	4.79 ^a^ ± 0.53	116.14 ^a^ ± 13.26	50 ^a^ ± 11.49
Group (2)	9.37 ^d^ ± 0.98	176.43 ^b^ ± 11.28	128.57 ^c^ ± 5.47
Group (3)	6.64 ^c^ ± 0.25	124 ^a^ ± 6.22	76.86 ^b^ ± 7.40
Group (4)	5.71 ^b^ ± 0.47	122.14 ^a^ ± 5.64	57.43 ^a^ ± 5.62
F-ratio	72.01	58.91	142.04
*p*-value	0.00	0.00	0.00

The different letters indicate statistically different means according to Duncan multiple range test.

**Table 4:** Effect of grape seed extract on hepatic catalase activity, GSH, and total protein content in albino rats injected with dexamethasone (*n* = 7).

Groups	Catalase (UI/mg Protein)	GSH (μg/mg Protein)	Total Protein (g/dL)
Group (1)	0.88 ^d^ ± 0.05	3.77 ^d^ ± 0.37	2.78 ^d^ ± 0.11
Group (2)	0.42 ^a^ ± 0.04	1.03 ^a^ ± 0.19	1.78 ^a^ ± 0.13
Group (3)	0.54 ^b^ ± 0.03	1.66 ^b^ ± 0.18	2.11 ^b^ ± 0.10
Group (4)	0.70 ^c^ ± 0.07	2.44 ^c^ ± 0.18	2.44 ^c^ ± 0.12
F-ratio	111.01	166.31	97.77
*p*-value	0.00	0.00	0.00

The different letters indicate statistically different means according to Duncan multiple range test. GSH is reduced form of glutathione.
